# Uric acid and obesity-related phenotypes in postmenopausal women

**DOI:** 10.1007/s11010-017-3215-6

**Published:** 2017-10-26

**Authors:** B. Grygiel-Górniak, M. Mosor, J. Marcinkowska, J. Przysławski, J. Nowak

**Affiliations:** 1 Department of Rheumatology and Internal Diseases, Poznan University of Medical Sciences, Poznan, Poland; 2 Department of Computer Science and Statistics, Poznan University of Medical Sciences, Poznan, Poland; 3 Department of Molecular Pathology, Institute of Human Genetics, Polish Academy of Sciences, Poznan, Poland; 4 Department of Bromatology and Human Nutrition, Poznan University of Medical Sciences, Poznan, Poland

**Keywords:** Uric acid, Pro12Ala and Trp64Arg polymorphisms, Postmenopausal women, Nutrition

## Abstract

The aim of this study was to find the genetic, metabolic, and nutritional risk factors, which can be associated with uric acid (UA) level. The risk factors related to uricemia were assessed among 271 postmenopausal women without cardiometabolic disorders and hypolipidemic/hypoglycemic treatment selected from a cohort of 1423 obese postmenopausal women. The bioimpedance analysis and biochemical and genetic analyses were performed in two groups characterized by serum UA ≥ 4 mg/dL (238 μmol/L) and < 4 mg/dL. The TaqMan-based real-time PCR method was applied to assess the role of Pro12Ala of peroxisome proliferation-activated receptor (PPAR)gamma-2 and Trp64Arg of beta-3-adrenergic receptor (ADRB) polymorphisms. Women with UA level ≥ 4 mg/dL were characterized by larger body mass, triceps skinfold, waist circumference, body fat amount, and serum insulin, glucose, and triglyceride levels. There was no difference in dietary habits between the analyzed groups. Body mass, waist circumference, body fat amount, diastolic blood pressure, and serum insulin, glucose, high-density lipoprotein, and triglyceride levels, Homeostasis Model Assessment-Insulin Resistance, and energy from the dietary fat influence the UA level ≥ 4 mg/dL; however, the serum UA was not determined by Pro12Ala and Trp64Arg polymorphism analyses. The model of linear regression revealed that the group characterized by body mass index  ≥ 25 kg/m^2^ and glucose ≥ 100 mg/dL has 4 times increased risk of UA level (*p* = 0.0009); after adding triglycerides ≥ 150 mg/dL, the risk of UA increased 7 times (*p* = 0.0216). Increasing the level of UA ≥ 4 mg/dL is associated with overweight, hyperglycemia, and hypertriglyceridemia in women without a history of cardiometabolic disorders. A better management of metabolic factors could help prevent further increase in UA levels.

## Introduction

More than 450,000 women suffer from cardiovascular diseases (CVD) annually, and more than half of them die of coronary artery disease [1]. The risk of heart disorders increases after the menopause and is associated with hormonal and metabolic changes [2]. The factor, which contributes to the high CVD risk, is elevated uric acid (UA) level [3–7], which represents an indirect marker of the metabolic syndrome [8, 9]. Hyperuricemia is related to obesity [10], diabetes mellitus [11], dyslipidemia [12, 13], and cancerogenesis [14]. However, no study has estimated such a risk in postmenopausal women without metabolic syndrome, diabetes mellitus, and severe CVDs.

Not only metabolic disorders, but also genes responsible for insulin resistance and obesity such as PPAR gamma 2 and ADRB3 may contribute to the development of hyperuricemia. The Pro12Ala polymorphism of PPAR has been associated with obesity [15], insulin sensitivity and diabetes [16–18], as well as lipid disorders [19]. However, the association of this genotype with UA is not well established [20, 21]. Another obesity-related phenotype, which participates in the regulation of lipolysis and thermogenesis is Trp64Arg polymorphism of ADRB [22]. The Arg64 allele is associated with dyslipidemia, glucose disorders, and elevated UA levels [22–24]. Moreover, carriers of the Arg64 allele who were normouricemic at the baseline were found to have a higher risk of developing hyperuricemia 6 years later [23].

To estimate the direct role of UA in the development of cardiometabolic disorders, different levels of UA are discussed depending on the oxidative and metabolic states [11, 25]. If serum antioxidants are used and an increased activity of oxidative processes is present in the atherosclerotic plaque, UA works as a prooxidative factor when its serum level is more than 4 mg/dL (particularly in obese people) [11]. At this level, UA inhibits the proliferation and migration of endothelial cells, causes dysfunction of the endothelium, and gives the background of a progression of atherogenic processes [11, 25]. Considering a strictly selected group of postmenopausal women (without essential metabolic and cardiologic disorder), we intended to clarify an association between UA level with the Pro12Ala and Trp64Arg polymorphisms, anthropometric parameters, metabolic disorders (glucose and lipid changes), and dietary habits in postmenopausal women.

## Materials and methods

### Analyzed group

Preliminary studies were carried out in a group of 1423 obese postmenopausal women. From this group, we selected 271 women who did not use hormone replacement therapy, lipid lowering, and hypoglycemic treatment or dietary supplements. We excluded women diagnosed with endocrinopathies significantly affecting the metabolism (hyper- or hypothyroidism and diabetes mellitus), metabolic syndrome, CVDs (with the exception of hypertension), and cancer and smokers.

### Anthropometric measurements

Height and weight were measured in underwear using the SECA scale. Waist circumference was determined at the narrowest point between the costal margin and the iliac crest, and the hip circumference was measured at the widest point over the buttocks. Body mass index (BMI) was calculated as weight in kilograms divided by height in meters squared. Waist-to-hip ratio (WHR) was estimated as the proportion of waist-to-hip circumferences [26]. A bioimpedance analyzer (Bodystat 1500, Bodystat Ltd, UK, a single frequency—50 kHz device) was used to assess fat content as the proportion of total body mass. Systolic (SBP) and diastolic (DBP) blood pressures were measured using a standard mercury sphygmomanometer in sitting position after taking a 10-min break in accordance with the guidelines of the European Society of Hypertension Working Group on BP monitoring [27].

### Nutritional evaluation

The dietary intake was assessed by the dietary recall through last 24 h over 7 consecutive days [28] and calculated from tables of composition and nutritional values of food products [29]. The dietary assessment was made by calculation of the intake of food components, and analyses were done after comparison with the recommended levels [26, 30].

### Biochemical analysis

Considering the evidence that the risk of atherosclerotic plaque development and prooxidative activity of UA is observed at the level ≥ 4 mg/dL [11], we divided the women into two groups with higher (≥ 4 mg/dL) and lower (≤ 4 mg/dL) UA levels. To check if there is a relation among metabolic parameters, genetic background, and UA levels, we performed a biochemical analysis in the restrictively selected postmenopausal women without metabolic diseases and severe CVDs. Blood samples were drawn after a minimum night fasting period of 12 h. Laboratorial analyses were performed using standard methodologies. UA, glucose, and lipid profile were analyzed with enzymatic colorimetric assays (Cobas Integra 400 Plus; Roche Diagnostics), follicle-stimulating hormone (FSH) via specific chemiluminescence assays from Roche Diagnostic, and plasma insulin using an enzymatic immunoassay (Cobas Integra 400 Plus; Roche Diagnostics). The serum level of low-density lipoproteins (LDL) was calculated using the formula of Friedewald et al. [31]. Homeostatic model assessment index was used to estimate insulin resistance [32]:${\text{HOMA1-IR}} = \left[ {{\text{fasting insulin }}\left( {\upmu {\text{U}}/{\text{mL}}} \right) \times {\text{fasting glucose}}\left( {\text{mM}} \right)} \right]/ 2 2. 5.$

Hyperuricemia is defined as the serum UA level exceeding 6.8 mg/dL (404 μmol/L) and at this concentration the crystallization of monosodium urate is observed (at physiological pH and body temperature) [33]. But the solubility of UA significantly diminishes at low pH and body temperature and crystallization of monosodium urate is indicated at 4 mg/dL (238 μmol/L) at 30 °C in less warmed and poorly vascularized (tendons, ligaments) or non-vascularized cartilages [34]. In the literature, many diverse arbitral borders of UA level have been suggested to be associated with oxidative stress and CVDs [11, 23]. The meta-analysis of Braga et al. showed that the threshold that identifies the level of UA at which the risk of CVDs escalates on a follow-up ranges from 5.3 to 7.7 mg/dL [11], whereas Morcillo et al. suggests a threshold of 6 mg/dL for women [23]. However, if the serum antioxidants are used and the increased activity of oxidative processes is present in the atherosclerotic plaque, mainly in obese people, UA is considered as a prooxidative factor at the serum level ≥ 4 mg/dL [11]. Because we analyzed specifically a selected group of postmenopausal women without metabolic diseases and severe CVDs, we considered 4 mg/dL of UA as a borderline level and intended to check if there is an association between the UA level and genetic and nutritional disorders in the analyzed group.

### Genetic parameters

#### Genotyping

Genomic DNA was isolated from venous blood samples, according to the manufacturer’s protocol (Gentra Puregene Blood Kit, Qiagen, Germany). Genotypes of the Pro12Ala (rs1801282) and Trp64Arg (rs4994) polymorphisms were determined by a TaqMan genotyping assay (Life Technologies, Carlsbad, California, USA). As a quality control measure, negative controls and approximately 5% of samples were genotyped in duplicate to check genotyping accuracy. The controls for each of the genotypes of the both SNPs were run in parallel. An allelic discrimination assay was performed on an ABI7900HT or on a CFX96 Touch Real-Time PCR Detection System (Bio-Rad, Hercules, California, USA). The genotypes were determined as Trp64Trp, Trp64Arg, and Arg64Arg without prior knowledge of the subjects’ status.

### Linkage disequilibrium (LD), block determination, and haplotype construction

The genotype data were used to construct the haplotypes between the two polymorphisms using the Haploview 4.2 software to evaluate LD. LD between the single-nucleotide polymorphisms (SNPs) used in haplotype analysis was measured using pairwise *D*′ statistics. The structure of the LD block was examined with the method proposed by Gabriel et al. using the 80% confidence bounds of *D*′ [35]. The haplotype frequencies were calculated based on the maximum likelihood method using Haploview 4.2 software. Finally, the associations between haplotypes and the obesity status were checked. Specific haplotype frequencies were compared among lean and obese women (Chi-square test).

### Statistical analysis

Allele and genotype frequencies were calculated, and odds ratio (OR) and the corresponding 95% confidence intervals (CI of the risk of higher UA levels) were estimated. We used a Chi-square test to determine whether the polymorphisms were in Hardy–Weinberg equilibrium in the population. Fisher’s exact test for an *R* × *C* table (Fisher–Freeman–Halton test) was used to estimate the association between selected polymorphisms and the risk of higher UA level. Continuous variables were verified according to the consistency with the normal distribution, using the Shapiro–Wilk test. If the data passed the normality test, we used parametric Student’s t test to compare the two groups, and if not non-parametric Mann–Whitney *U* test was used. Logistic regression models were used to find the selected factors, which may contribute to higher UA levels (≥ 4 mg/dL). We calculated the coefficient of multiple determination *R* ^2^ using the Cox–Snell equivalent of the coefficient of multiple determination *R* ^2^ defined for multiple linear regression. After adding to this model genetic variable (such as Pro12Ala or Trp64Arg), a particular *p* value of the likelihood ratio was calculated to compare the analyzed logistic regression models. A *p* value of < 0.05 was considered statistically significant, and all reported *p* values were two-tailed. The statistical analysis was performed using Statistica v. 12.0 (StatSoft, Inc.).

## Results

Both groups of women were of similar ages and had similar heights (Table 1). Women characterized by ≥ 4 mg/dL UA level have higher body mass, triceps skinfold, waist circumference, body fat amount, diastolic blood pressure, serum insulin, glucose, and triglyceride levels, lower HDL level, and higher calculated WHR, HOMA-IR, and atherogenic indexes (LDL/HDL and TG/HDL). The analysis of dietary habits in both groups showed that daily diets had approximate amounts of energy of 2000 kcal. The percentage of energy from protein was about 16%, that from fat ranged from 32% (low UA group) to 34% (high UA group), whereas that from carbohydrates exceeded 50% in both groups. Nearly 12% of energy in daily food rations was from saturated fatty acids (SFA). The proportion of energy from monounsaturated fatty acids (MUFA) was about 13%, whereas the percentage of energy from polyunsaturated fatty acids (PUFA) exceeded 5% of energy in both the analyzed groups.

**Table 1:** Anthropometric, biochemical, and nutritional characteristics of the analyzed groups with low and high serum UA levels

Analyzed parameters	Low UA < 4 mg/dL; *X* ± SD; *n* = 58	High UA < 4 mg/dL; *X* ± SD; *n* = 213	*p* value
Age (years)	59.67 ± 6.23	59.21 ± 5.24	0.5695
Height (cm)	160.92 ± 5.41	160.95 ± 5.93	0.9773
Body mass (kg)	65.59 ± 12.36	79.30 ± 16.06	0.00001
BMI (kg/m^2^)	25.0 ± 4.42	30.62 ± 6.23	0.00001
Triceps skinfold (mm)	16.05 ± 4.37	20.72 ± 6.13	0.00001
Waist circumference (cm)	80.47 ± 11.43	93.08 ± 13.82	0.00001
WHR	0.80 ± 0.08	0.85 ± 0.07	0.0002
Body fat (%)	39.07 ± 5.97	44.68 ± 6.42	0.00001
Systolic blood pressure (mmHg)	136.67 ± 23.04	142.47 ± 22.27	0.0822
Diastolic blood pressure (mmHg)	84.12 ± 12.75	89.03 ± 13.63	0.0143
FSH (mIU/ML)	75.04 ± 27.39	68.71 ± 25.95	0.1051
Insulin (mU/dL)	6.99 ± 3.79	10.42 ± 8.08	0.0019
Glucose (mg/dL)	91.66 ± 8.49	97.89 ± 14.36	0.0017
HOMA-IR	1.62 ± 1.00	2.59 ± 2.14	0.0009
TC (mg/dL)	228.24 ± 42.22	231.59 ± 40.68	0.5819
HDL (mg/dL)	70.14 ± 14.85	62.32 ± 14.24	0.0002
TG (mg/dL)	93.73 ± 46.95	123.96 ± 53.92	0.0001
LDL (mg/dL)	139.35 ± 37.15	144.49 ± 36.62	0.3459
LDL/HDL	2.09 ± 0.79	2.43 ± 0.79	0.0040
TG/HDL	1.48 ± 1.05	2.18 ± 1.29	0.0002
UA (mg/dL)	3.49 ± 0.35	5.18 ± 0.92	0.00001
Energy (kcal)	2009.81 ± 592.76	2060.32 ± 548.98	0.5419
Protein % energy	16.17 ± 3.48	16.34 ± 3.24	0.7328
Fat % energy	32.95 ± 6.01	34.33 ± 5.06	0.0785
Carbohydrate % energy	51.64 ± 7.57	50.20 ± 6.46	0.1467
SFA % energy	11.98 ± 2.88	12.14 ± 2.29	0.6513
MUFA % energy	12.56 ± 2.92	13.36 ± 2.73	0.0504
PUFA % energy	5.42 ± 1.75	5.76 ± 1.75	0.1911

*UA* uric acid, *X* mean, *SD* standard deviation, *n* number of patients, *WHR* waist-to-hip ratio, *TC* total cholesterol, *HDL* high-density cholesterol, *TG* triglycerides, *LDL* low-density cholesterol, *SFA* saturated fatty acids, *MUFA* monounsaturated fatty acids, *PUFA* polyunsaturated fatty acids

The frequency of Trp64 alleles was higher in the group, with UA ≥ 4 mg/dL (Table 2). The impact of selected anthropometric, biochemical, and nutritional factors with adjustment for analyzed polymorphisms on the UA level (estimated by logistic regression models) showed that adding Pro12Ala or Trp64Arg polymorphism did not influence the UA level (Table 3). In the model of linear regression, the group characterized by overweight and hyperglycemia (BMI ≥ 25 kg/m^2^ and glucose ≥ 100 mg/dL) had 4 times higher risk of high UA levels (4.352; Fisher’s exact test *p* = 0.0009). Moreover, after adding the third variable such as triglyceride (≥ 150 mg/dL) to this model, the risk of high UA level increased nearly 7 times (7.579; Fisher’s exact test *p* = 0.0216).

**Table 2:** Genotype and allele frequencies of the Prol2Ala of PPAR gamma-2 and Trp64Arg of beta-adrenergic receptor gene polymorphisms according to low uremic (UA < 4 mg/dL) and high uremic states (UA ≥ 4 mg/dL)

Analyzed genotypes	UA < 4 mg/dL *n* = 58	UA ≥ 4 mg/dL *n* = 213	Whole group of postmenopausal women *n* = 271
Genotype			
Pro12Pro	43 (74.14%)	142 (66.67%)	185 (68.27%)
Pro12Ala	14 (24.14%)	62 (29.11%)	76 (28.04%)
Ala12Ala	1 (1.72%)	9 (413%)	10 (3.69%)
Chi square *p* value	0.5179		
Allele frequency			
Pro	100 (86.21%)	346 (81.22%)	446 (82.29%)
Ala	16 (13.79%)	80 (18.78%)	96 (17.71%)
Chi square *p* value	0.2124		
OR (95% CI)	1.4451 (0.8082; 2.5837)		
Genotype			
Trp64Trp	44 (75.86%)	176 (82.63%)	220 (81.18%)
Trp64Arg	12 (20.69%)	35 (16.43%)	47 (17.34%)
Arg64Arg	2 (3.45%)	2 (2.94%)	4 (1.28%)
Chi square *p* value	0.1823		
Allele frequency			
Trp	100 (86.21%)	387 (94.16%)	487 (89.85%)
Arg	16 (13.79%)	24 (5.84%)	55 (10.15%)
Chi square *p* value	0.0043		
OR (95% CI)	0.3876 (0.1984; 0.7572)		

Data are tabulated in terms of *n* (%) for genotypes and n (frequency) for alleles

*Prol2Ala* polymorphism of PPAR gamma-2 gene, *Trp64Arg* polymorphism of ADRB3 gene, *OR* odds ratio, *CI* confidence interval

**Table 3:** Impact of selected factors on the UA level > 4 mg/dL with adjustment for the analyzed polymorphisms in logistic regression models

Analyzed parameters	Statistical analysis without polymorphisms				Statistical analysis after adding polymorphisms			
					Pro12Ala		Trp64Arg	
	OR	95% CI	*p* value	*R* ^2^ (%)	*p* value	*R* ^2^ (%)	*p* value	*R* ^2^ (%)
Body mass (kg)	1.07	1.04–1.1	< 0.000001	13.1	< 0.000001	13.4	< 0.000001	13.8
Triceps skinfold (mm)	1.15	1.09–1.22	< 0.000001	10.0	< 0.000001	10.4	< 0.000001	10.5
Waist circumference (cm)	1.08	1.05–1.11	< 0.000001	13.7	< 0.000001	13.9	< 0.000001	14.5
Body fat (%)	1.15	1.09–1.22	< 0.000001	11.8	< 0.000001	12.2	< 0.000001	12.3
Diastolic BP (mmHg)	1.03	1.01–1.05	0.01	2.3	0.0220	2.8	0.0228	2.8
BMI (kg/m^2^)	1.22	1.14–1.31	< 0.000001	14.9	< 0.000001	15.2	< 0.000001	15.4
FSH (mIU/ML)	0.99	0.98–1.0	0.11	1.0	0.1408	1.4	0.1533	1.4
Insulin (mU/dL)	1.16	1.07–1.26	0.001	6.3	0.0001	6.8	0.0001	7.0
Glucose (mg/dL)	1.05	1.02–1.08	0.0006	4.3	0.0009	5.0	0.0011	4.9
HDL (mg/dL)	0.97	0.95–0.98	0.0004	4.5	0.0009	5.0	0.0011	4.9
TG (mg/dL)	1.02	1.01–1.02	0.00002	6.6	0.0001	6.9	0.0000	7.3
HOMA-IR	1.82	1.31–2.52	0.00001	6.9	0.00003	7.4	0.00002	7.6
Protein % energy	1.01	0.99–1.03	0.19000	0.6	0.23514	1.1	0.19779	1.2
Fat % energy	1.15	1.09–1.22	< 0.000001	11.8	< 0.000001	12.2	< 0.000001	12.3
Carbohydrate % energy	0.97	0.92–1.01	0.14159	0.8	0.16071	1.3	0.15845	1.4

*p* value of the likelihood ratio test, *R* ^*2*^ the Cox–Snell equivalent of the coefficient of multiple determination *R* ^2^ defined for multiple linear regression, *FSH* follicle-stimulating hormone, *BP* blood pressure

## Discussion

Menopause is a risk factor for CVDs associated with hormonal and metabolic changes [1, 2]. Increased body fat mass, particularly viscerally distributed, insulin resistance, blood pressure, and lipid disorders contribute to cardiometabolic disorders [36–38]. In the literature, the measurement of serum UA as a diagnostic criterion to define the metabolic syndrome is still under discussion; moreover, the borderline of this parameter is differently interpreted in many studies [11, 39]. Taking into account the fact that in obese people UA is considered as a prooxidative factor at the serum level ≥ 4 mg/dL [11], we considered this concentration of UA as a borderline level and plan to check an association between the UA level and genetic and nutritional disorders in the analyzed group.

The analysis of data presented in Table 1 showed that postmenopausal women with UA ≥ 4 mg/dL were characterized by visceral obesity (BMI ≥ 30 kg/m^2^, waist circumference > 93 cm, WHR ≥ 0.85) and higher serum glucose, insulin, and lipoprotein levels. However, in both groups the insulin, glucose, and triglyceride levels were within the recommended level and HOMA-IR values did not cross 3. These data can be explained by the fact that women in this study were selected according to strict criteria. We calculated the risk of CVDs Systematic Coronary Risk Estimation—SCORE, which, according to the European Society of Cardiology criteria, was within the range 1–5% [40] and, thus, the levels of total cholesterol < 190 mg/dL and LDL < 115 mg/dL were considered as recommended in the analyzed groups [40, 41]. Using these criteria, hypercholesterolemia was newly diagnosed; however, there were no differences in the total cholesterol and LDL levels between the analyzed groups.

The nutritional analysis shows that the energy value satisfied the recommended level; however, the daily food rations in both groups were improperly balanced. The protein content exceeded 16% of energy (the recommended intake for the Polish population is 10–15% of energy from diet) [30, 42] and this was associated with an increased percentage of energy from fat (exceeding 30% of energy), which may unfavorably influence serum glucose and lipid levels and increase the risk of cardiovascular disorders [43, 44]. The intake of carbohydrates was low and did not satisfy the recommended energy from this component, which should be about 55–75% (particularly unrefined carbohydrate characterized by a low glycemic index) [30, 42].

Since the type of fatty acids consumed is more important than the total quantity of fat when looking at metabolic goals and CVD risk [45, 46], we analyzed the intake of selected fatty acids. The intake of saturated fatty acids was high (exceeding the recommended intake of 8–10% of energy), which is associated with increased cardiovascular risk [45–47]. The proper amount of monounsaturated fatty acids should range from 13 to 14%, whereas polyunsaturated acids should account for about 8 to 9% of energy in daily food rations [30]. Moreover, multiple randomized controlled trials have reported that a Mediterranean-style eating pattern rich in monounsaturated fats can improve both glycemic control and blood lipids [45, 48, 49]. In this study, a comparatively high content of monounsaturated acids was observed, but this was associated with a low intake of PUFA. Consuming polyunsaturated fat in place of saturated fat should be advised, because it reduces the risk of coronary heart disease [50] and, thus, should be advised for the analyzed groups of women.

From the results of the Chi-square test, the distribution of selected genotypes and allele frequencies showed no differences between Pro12Ala polymorphisms and allele distribution in the analyzed groups (Table 2). The prevalence of Pro12Ala polymorphism and Trp64Arg was similar as in other Caucasians [22, 51, 52]. We did not find any difference between the frequencies of the selected Trp64Arg genotypes; however, the frequencies of Trp allele and Arg allele were statistically significant. The Arg64 allele predisposes to higher body fat and serum glucose [22] and elevated serum UA levels [22, 53], and predicts a greater tendency to develop abdominal adiposity and high blood pressure with an advancing age [54]. However, in this study we did not find such associations, and participants with Trp64Trp or Arg64X genotypes (including Trp64Arg and ARg64Arg variants) were found to have similar metabolic parameters (including UA level) and the daily food rations were comparable (data not shown in tables).

In the logistic regression models (Table 3), we estimated that many of the analyzed factors (body mass, triceps skinfold, waist circumference, body fat amount, diastolic blood pressure, BMI, serum insulin, glucose, HDL, and triglyceride levels, HOMA-IR, and energy from dietary fat) determined the UA level ≥ 4 mg/dL. The majority of these parameters are described as risk factors of metabolic syndrome development [8–11]. However, none of the analyzed groups met the criteria of metabolic syndrome according to the International Diabetes Federation (at least three of the five risk factors: waist circumference ≥ 80 cm or BMI ≥ 30 kg/m^2^; TG ≥ 150 mg/dL; HDL < 50 mg/dL; fasting glucose ≥ 100 mg/dL; SBP ≥ 130 mmHg, DBP ≥ 85 mmHg, and/or specific treatment) [8, 55]. In women with UA ≥ 4 mg/dL, visceral obesity, hypertension, and hypercholesterolemia were observed, but the average concentrations of HDL, TG, and glucose were within the recommended norms. Adding new variables (Pro12Ala and Trp64Arg polymorphisms) to logistic regression models did not explain the level of UA any better.

The model of linear regression was used to find which of the metabolic parameters might influence a higher level of UA (Table 4). This model shows that the group characterized by overweight and hyperglycemia has 4 times increased risk of UA higher than 4 mg/dL, but after adding serum triglycerides risk augments 7 times. In the literature, high serum concentration of UA is considered as a risk factor of CVDs or metabolic disorders; however, higher concentrations of this parameter have been considered [3–7, 37, 38]. Thus, overweight women with hyperglycemia and hypertriglyceridemia have a higher chance of increasing UA level. We suspect that the analysis of women with metabolic disorders and CVDs might show stronger associations of the discussed parameters and, particularly, that the risk of CVDs markedly increases when serum uric acid UA concentration is  >  7.0 mg/dL (420 μmol/L) [11]. Moreover, the UA concentration rises with age [56] and augments acutely after the ingestion of fructose [57]. Besides this, UA is elevated in serum non-alcoholic fatty liver disease and gout [58, 59] and has a causal role in metabolic syndrome and cardiovascular diseases [60]. Considering these facts, the prospective study may show stronger associations of metabolic parameters with elevated cardiometabolic disorders in the analyzed group.

**Table 4:** Risk assessment of UA levels in groups with various metabolic disorders

Analyzed groups	UA < 4 mg/dL *n* = 58	UA ≥ 4 mg/dL *n* = 213	Whole group of postmenopausal women *n* = 271
Risk group (BMI ≥ 25 kg/m^2^ and glucose ≥ 100 mg/dL)	5 (8.6%)	62 (29.1%)	67 (24.7%)
Non-risk group	53 (91.4%)	151 (70.9%)	204 (75.3%)
Fisher’s exact test (*p* value)	0.000962		
OR (95% CI)	4.3523(1.6607; 11.4058)		
Risk group (BMI ≥ 25 kg/m^2^ and glucose ≥ 100 mg/dL and TG150 mg/dL)	1 (1.72%)	25 (11.74%)	5 (9.59%)
Non-risk group	57 (98.28%)	188 (88.26%)	245 (90.40%)
Fisher’s exact test (*p* value)	0.021567		
OR (95% CI)	7.5797 (1.0048; 57.1748)		

The prevalence of hyperuricemia is higher among people with increased cardiovascular risk, including postmenopausal women (estrogens promote renal excretion of uric acid), obese people, hypertension, diabetes mellitus, and dyslipidemia [61, 62]. The women in the analyzed group were selected based on the absence of cardiovascular disorders, but they were of postmenopausal age and characterized by obesity, dyslipidemia, and hypertension. Thus, in this group the cardiovascular risk is high and the metabolic parameters of postmenopausal women should be monitored, including the UA level.

## Conclusions

In the literature, many authors show the relationship between high uric acid levels (> 6 mg/dL) and the prevalence of metabolic disorders in numerous diseases such as cardiovascular diseases, diabetes mellitus, gout, or non-alcoholic fatty liver disease. This study shows that even at low uric acid levels metabolic disorders are present such as obesity, dyslipidemia, and hypertension. Besides this, we showed that overweight, hyperglycemia, and hypertriglyceridemia are predictors of higher UA levels and thus metabolic parameters should be carefully monitored for the development of metabolic disorders and CVD in every woman of postmenopausal age. Heterozygous mutation of Trp64Arg in the *beta3*-*AR* gene may partly contribute to the accumulation of multiple risk factors in postmenopausal obese women with UA level ≥ 4 mg/dL. Even at low serum uricemia, special attention should be paid to nutritional habits and the diet should be modified to not only treat obesity, dyslipidemia, and hypertension but also prevent the increase of uric acid in the future.
